# Mapping the Brain’s electric fields with Magnetoelectric nanoparticles

**DOI:** 10.1186/s42234-018-0012-9

**Published:** 2018-08-06

**Authors:** R. Guduru, P. Liang, M. Yousef, J. Horstmyer, S. Khizroev

**Affiliations:** 1 Center for Personalized Nanomedicine, Florida International University, 11200 SW 8th ST, Miami, Florida 33199 USA; 2 Department of Electrical and Computer Engineering, Florida International University, Miami, Florida 33174 USA; 3 Department of Electrical and Computer Engineering, University of California, Riverside, California, 92521 USA; 4 Brain Center, Miami, Florida 33124 USA

**Keywords:** Nanotechnology, Brain mapping, Magnetoelectric, Nanoparticles, Magnetic particle imaging, Reverse engineering the brain

## Abstract

**Background:**

Neurodegenerative diseases are devastating diagnoses. Examining local electric fields in response to neural activity in real time could shed light on understanding the origins of these diseases. To date, there has not been found a way to directly map these fields without interfering with the electric circuitry of the brain. This theoretical study is focused on a nanotechnology concept to overcome the challenge of brain electric field mapping in real time. The paper shows that coupling the magnetoelectric effect of multiferroic nanoparticles, known as magnetoelectric nanoparticles (MENs), with the ultra-fast and high-sensitivity imaging capability of the recently emerged magnetic particle imaging (MPI) can enable wirelessly conducted electric-field mapping with specifications to meet the requirements for monitoring neural activity in real time.

**Methods:**

The MPI signal is numerically simulated on a realistic human brain template obtained from BrainWeb, while brain segmentation was performed with BrainSuite software. The finite element mesh is generated with Computer Geometry Algorithm Library. The effect of MENs is modeled through local point magnetization changes according to the magnetoelectric effect.

**Results:**

It is shown that, unlike traditional magnetic nanoparticles, MENs, when coupled with MPI, provide information containing electric field’s spatial and temporal patterns due to local neural activity with signal sensitivities adequate for detection of minute changes at the sub-cellular level corresponding to early stage disease processes.

**Conclusions:**

Like no other nanoparticles known to date, MENs coupled with MPI can be used for mapping electric field activity of the brain at the sub-neuronal level in real time. The potential applications span from prevention and treatment of neurodegenerative diseases to paving the way to fundamental understanding and reverse engineering the brain.

## Background

It is difficult to overestimate the significance of the capability to map intrinsic electric fields induced by neural activity deep in the brain with adequately high spatial and temporal resolutions to monitor this activity in real time (Fox & Raichle, 2007; Marblestone et al., 2013). The potential applications span from prevention and treatment of neurodegenerative diseases to paving the way to fundamental understanding and reverse engineering the brain (Koch & Reid, 2012). This paper presents a theoretical study to exploit a nanotechnology solution for addressing this challenge. To underscore the significance of the novel concept, the discussion of the study is preceded by a brief overview of the current state of the art.

### State of the art

Functionalized brain imaging aims to study basic mechanisms of electric-field-driven cognitive processes (Cabeza & Nyberg, 2000; Pascual-Marqui, 2002). In spite of significant advances in this field especially during the last two decades, the existing technologies for recording neural activity are severely limited in their capabilities. These technologies include electroencephalography (EEG) (Coenen, 1995), functional magnetic resonance imaging (fMRI) and diffusion MRI (dMRI), also known as diffusion tensor imaging (DTI), or a combination of these two (DfMRI) (Yassa et al., 2010), positron emission tomography (Lee et al., 2012; Grafton et al., 1992), magnetoencephalography (MEG) (de Pasquale et al., 2010), neuronal optogenetics (Toettcher et al., 2010), molecular recording (Zamft et al., 2012), and others. Brain imaging with a spatial resolution of 1 mm can be achieved non-invasively with the MRI approaches; however, these they mostly provide a structural map and only indirectly and with a limited accuracy reflect the electric field perturbations due to neural activity; their temporal resolutions are limited by the hemodynamic response to approximately 1 s. To detect the neural activity through the hemodynamic response, fMRI and dMRI use the blood-oxygen-level dependent (BOLD) contrast and the contrast based on the strength of the diffusion of water molecules, respectively. PET, with a comparable spatial resolution of 1 mm, can be used to monitor the brain metabolism and neurochemistry; like the MRI approaches, this technique only indirectly depends on the local electric field and is limited only to the processes which can be observed with radioactively labeled organic molecules. MEG can detect a magnetic field induced by the small electric currents due to neural activity; however, this approach is limited by both the complexity of the sensor technology required to detect the extremely weak stray magnetic field (~ 10 fT) above the skull and the difficulty of solving the notorious inverse problem required for mapping the brain. EEG is capable of a sub-millisecond temporal resolution but requires the use of large arrays of electrodes and is 2D limited; moreover, like MEG, it deals with the same mathematical challenge of the inverse problem. Neuronal optogenetics is a relatively new and promising approach; however, it has too many open questions associated with the optical readout from many neurons (Deisseroth et al., 2006). Single-neuron level molecular recording has been proposed; however, this approach is still at its conceptual level (Zamft et al., 2012). Technical and fundamental limitations of these and other existing technologies are described in more detail elsewhere (Marblestone et al., 2013). In summary, for decades, progress in neuroimaging has significantly improved our understanding of the field dynamics in the brain; nevertheless, still there is no practical way to directly map local electric fields in response to neural activity in real time without interfering with the normal operation of the brain.

### Nanotechnology solution

Recently, a concept has been pioneered to exploit unique properties of magnetoelectric nanoparticles (MENs) (Eerenstein et al., 2006) to wirelessly access a local electric field activity deep in the brain for both wirelessly controlled local stimulation and mapping of neural activity (Yue et al., 2012; Guduru et al., 2015). Similar to traditional magnetic nanoparticles (MNs), MENs have a non-zero magnetic moment. Therefore, they can be remotely detected through a magnetic imaging approach and/or transported across the blood-brain barrier (BBB) and then to a desired target site(s) deep in the brain via application of a specially timed and image-guided sequence of magnetic field gradients (Nair et al., 2013). In addition, unlike MNs, MENs display a non-zero magnetoelectric (ME) effect. This effect, e.g., present in some type I multiferroics due to the relatively strong strain-related coupling between the ferroelectric and ferromagnetic components, can be explained thermodynamically according to the phenomenological Landau theory of multiferroics through the 2nd order cross-term of the free energy, G (Landau & Lifshitz, 1960):

1 $$ G\left(E,H\right)=-{\alpha}_{ij}{E}_i{H}_j, $$

where E_i_ and H_j_ stand for the i-th and j-th components of the local electric or magnetic fields, respectively, and α_ij_ represents the magnetoelectric tensor. As a result, in this approximation, the induced magnetization change of the nanoparticle depends on the local electric field according to the following linear expression:

2 $$ \varDelta {M}_i=-\partial G/\partial {H}_i={\alpha}_{ij}{E}_i. $$

For example, considering the value for *α* on the order of 0.1 G cm V^− 1^, a typical local electric field due to an action potential at the neuronal membrane on the order of 1 V/cm would induce a magnetization change of 1 emu/cc (Rodzinski et al., 2016; Guduru et al., 2013; McFadden, 2002; Stimphil & et al., 2017).. Assuming the MEN’s saturation magnetization is on the order of 10 emu/cc, the relative change in the magnetization on the order of 10% would be quite significant for a magnetic imaging technique to provide an adequate contrast. For example, if MENs are used instead of traditional MNs, e.g., superparamagnetic iron oxide nanoparticles (SPIONs), to enhance the image contrast, not only can they provide a structural image but also they can generate an electric field map.

Mapping the electric field deep in the brain at the cellular level could provide an important insight into our understanding of the brain. It is important to have a temporal resolution in the microsecond range or better to be able to record neural activity in real time. It is noteworthy that magnetic nanoparticles are used together with the traditional MRI system to enhance the image contrast. Unfortunately, MRI with a temporal resolution on the order of a second would not be adequately fast for the purpose of imaging in real time. A significant part of the neural activity takes place in a time domain on a millisecond scale or faster. Therefore, integration of MENs with the recently emerged approach known as magnetic particle imaging (MPI), which fundamentally provides a significantly faster detection rate compared to MRI, could pave a way to the next generation real-time neuroimaging (Gleich & Weizenecker, 2005; Goodwill et al., 2009; Weizenecker et al., 2009; Buzug and Borgert, 2012. Though in general the MPI setup might look similar to the traditional MRI system, the underlying physics is quite different in these two cases. For comparison, the MRI signal is enhanced through the shift of the nuclear spin relaxation times T1 and T2 by the nanoparticle-induced local magnetic fields and thus the temporal resolution of MRI is limited by the nuclear spin relaxation time. In contrast with MPI, the signal is independent of the nuclear relaxation times and instead is directly determined by the switching dynamic of the electron spin within the nanoparticle and therefore is limited by the ferromagnetic resonance time constant, which in turn is determined by the anisotropy energy of the nanostructure. For relatively high anisotropy magnetic nanostructures, the switching time can be in a sub-microsecond or even sub-nanosecond range. To select an image plane (section) in MPI, special selection coils, similar to the gradient coils in MRI, are used; however, the field generated by the selection coils is oppositely directed while comparable in magnitude to the uniform background field. This field ensures that the selected nanoparticles in the sectioned region are not magnetically saturated and thus can provide a relatively strong linear response to a small a.c. magnetic field. The a.c. frequency can be chosen in a wide range depending on the specific application requirements. For example, with MENs, the frequency can be chosen to maximize the magnetoelectric coupling, which is known to be frequency dependent (Nagesetti et al., 2017). Because of this different physics, MPI is supposed to provide orders of magnitude better sensitivity compared to the state-of-the-art MRI. The signal originates from the electron spin rather the nuclear spin (in MRI); the electron spin is approximately two thousand times larger than the nuclear spin. In addition, MPI doesn’t require either a high background field (in the Tesla range) or an extremely high field uniformity (of better than 1 ppm). For example, if MENs with a coercivity field of approximately 100 Oe are used, a background field of 500 Oe is sufficient to magnetically saturate the nanoparticles. The requirement on the background field uniformity is also quite relaxed. Indeed, the oppositely directed selection field of – 500 +/− 50 Oe could ensure that the moment of the selected particle is driven in the linear unsaturated region near zero field. Last but not least, the fundamental temporal resolution of MPI (< 1 μs) is superior to that of MRI (< 1 s).

In summary, if MENs are used instead of MNs together with MPI, they are expected to modulate the magnetic (structural) image with the local electric field due to the neural network activity with a 3D spatial resolution comparable to that of MRI or better and with a temporal resolution in the sub-microsecond range to meet the requirements for real-time monitoring of neural activity. Because of the use of nanoparticles, the spatial resolution can be eventually further improved through implementing advanced electromagnetic sources and signal processing and is fundamentally limited only by the nanoparticle size.

## Numerical methods

In this numerical computation, all the MPI signal simulations were performed on a realistic human brain template (using T1 and PD weighted phantom images) obtained from BrainWeb (Cocosco et al., 2003). Brain segmentation (WM, GM, and CSF) of the template was performed using BrainSuite software (Klauschen et al., 2009). The finite element (FE) mesh required for the segmented brain was generated using Computer Geometry Algorithm Library (CGAL) according to previously described method (Lee et al., 2012). The relative magnetization, M_rel_, defined as the ratio M/M_S_, where M_S_ is the saturation magnetization, was computed using standard magnetic dynamic formalism described elsewhere (Ivanov et al., 2007; Mikhaylova et al., 2004). The effect of the local electric field was modeled through the local point magnetization change according to the aforementioned linear expression for the ME effect. To estimate the change of the electric polarization, we assumed that local neural firings resulted in instantaneous currents density of 70 pA/pF (Tottene et al., 2002) and the electric field intensity was modeled considering the direct current conduction finite element method (FEM) (Miranda et al., 2012). To calculate the resulting electric field profiles, the system was divided into the following three segments, each with a uniform isotropic electric conductivity: (i) white matter (WM) – 0.14 S/m, (ii) grey matter (GM) – 0.33 S/m, (iii) cerebrospinal fluid (CSF) – 1.79 S/m (Wolters et al., 2006).

The following modeling parameters were used for 50-nm nanopartilces: the saturation magnetization, M_s_, of 10 and 100 emu/g for MENs and MNs, respectively, the isotropic ME coefficient, α (α_ij_ = α), of 0.1 G cm V^− 1^ for MENs, and the uniform microenvironment temperature, T, of 300 K. The basic physics of the MENs’ surface charge dependence on the field-dependent microenvironment was described in our previous publications (Guduru & Khizroev, 2014).

It was assumed that MENs could be administrated intravenously and then transported across BBB via application of a magnetic field gradient, as previously demonstrated through in vitro and in vivo studies (Guduru et al., 2015; Nair et al., 2013). By default, the modeled nanoparticle dose was approximately 0.1-mmol per 1 kg bodyweight or approximately 2.3 g of MENs for a 50-Kg weight subject. The concentration was comparable to the typical concentration of clinically used gadolinium based contrast agent (GBCA) (Voth et al., 2009). To illustrate the main concept, the effects of MENs on MPI-based brain imaging were studied on examples of relatively well-known neurocognitive responses that reflect specific functions in specific brain regions. MENs were compared to equivalent MNs. Again, the main distinction between MENs and MNs was the presence of the ME effect in MENs and its lack in MNs. As a result, the magnetic response of individual MENs depended not only on the magnetic moment but also on its surface charge; in contrast, the magnetic response of individual MNs did not depend on the surface charge. Assuming the nanoparticles were adequately separated from each other to exclude quantum-mechanical interactions between them (> 5 nm) but yet not too far (~ 1 μm) to ignore their collective effect due to the dipole-dipole interaction, their local collective behavior at the micron-size scale could be described by a mean-field theory of a paramagnetic gas using Langevin functions. The magnetic moment of each MN, *m*_*MN*_, was found as the product of its saturation magnetization, *M*_*S*_, and volume, *V*:

4 $$ {m}_{MN}={M}_SV $$

As discussed above, unlike MNs, MENs had an additional shift in their magnetization value due to the ME effect, ΔM_MEN_. In an isotropic approximation, the dependence of the magnetic moment shift on the averaged local electric field, E, was approximately given by the trivial linear expression:

5 $$ \varDelta {m}_{MEN}=\alpha EV $$

## Results

**Fig. 1:**
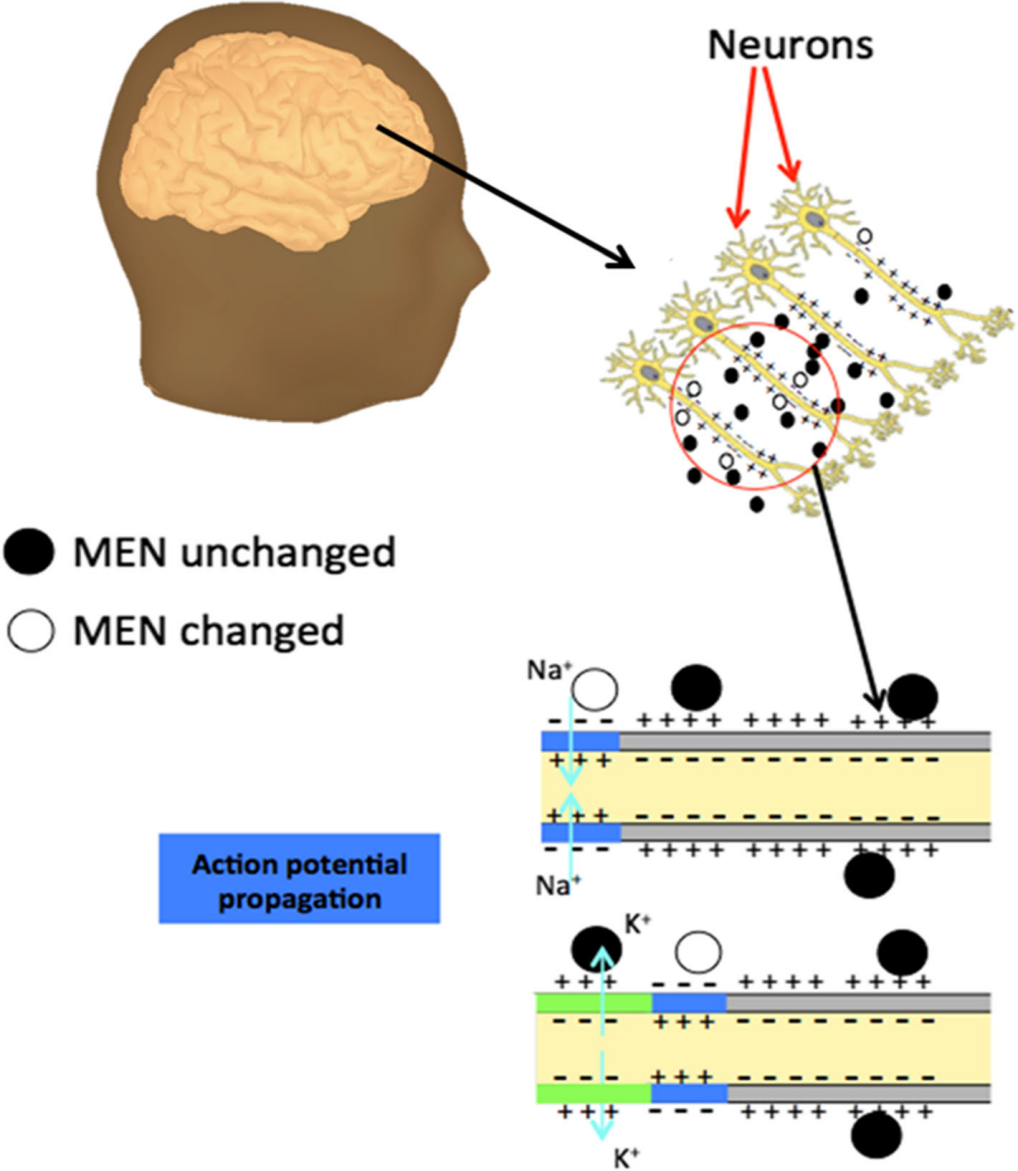
A schematic illustrating how MENs can be used to detect the electric field due to the neural activity deep in the brain. The exaggerated illustration shows how the magnetic moment of a MEN in the proximity of the membrane can be reversed by flipping the local electric field as the action potential travels by the MEN

Figure 1 illustrates how the MEN-based approach can be used to map the electric field in the brain. The main concept is illustrated on a popular example of an action potential travelling down an axon, which is consequently experiencing an electric polarity change across the membrane. The exaggerated illustration shows how the magnetic moment of a MEN near the membrane can be flipped by the reversed local electric field as the action potential travels by the MEN. The flipped magnetic moment and consequently the changed action potential can be detected through the resulting local contrast change in the magnetic image. It should be understood that this simplified example is used merely for the purpose of a conceptual illustration. In real applications, the complete 180-degree reversal (flipping) of the magnetic moment is not required as long as the change of the magnetic moment triggered by the local change of the electric field in a selected direction can be detected by the imaging system. Further, because the excitatory postsynaptic potentials (EPSPs) that are generated at the neuron’s apical dendritic tree last longer than the original action potentials, it is possible that they would make a significantly stronger contribution to the local electric field change and therefore would be easier to detect (compared to instantaneous action potentials). However, to succinctly describe the new fundamental concept we focus on the basic scenario of the instantaneous electric field change induced by an action potential. With the above, this paper presents a theoretical study to demonstrate how MPI using MENs could be used to read neuronal firing in the brain.

**Fig. 2:**
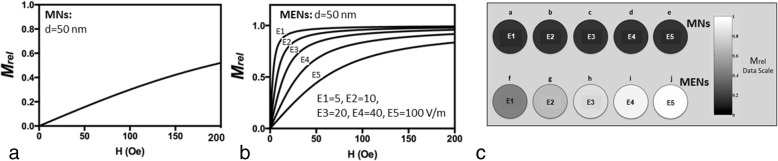
Magnetic field response of MNs and MENs depending on the nanoparticle size and local electric field at the micron-size scale. **a** M_rel_ (H) for MNs. (**b**) M_rel_ (H) for MENs for five different values of the local electric field: E1 = 5, E2 = 10, E3 = 20, E4 = 40, E5 = 100 V/m. The relative magnetization is the ratio of the magnetization and its saturated value

The locally averaged (at the micron scale) field-dependent magnetizations of individual 50-nm MNs and MENs are shown in Figs. 2a and b, respectively. No hysteresis is considered in this calculation. As described above, with MENs, the magnetic moment was affected by a local electric field. For example, the field dependences of the magnetization for five values of the local electric field, 5, 10, 20, 40, and 100 V/m, respectively, are shown in Fig. 2b. The field values were on the same order as endogenous electric fields deep in the brain. The relative magnetization was defined as the ratio of the magnetization and its saturated value. The calculated MPI signals for both MNs and MENs for the five electric field values are shown in Fig. 2c.

**Fig. 3:**
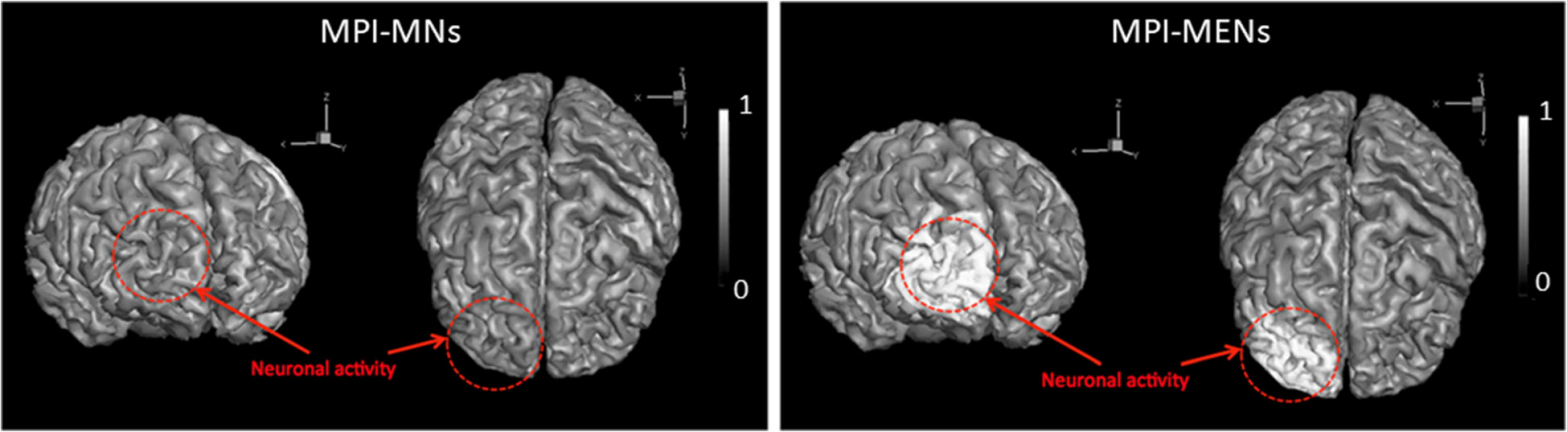
Normalized MPI images taken with MNs and (right) MENs of two different angle views of the frontal lobes of the cerebral cortex. The region with a simulated neuronal firing is highlighted by the dotted circle

For comparison, normalized MPI images enhanced with both MNs and MENs at two different view angles of the frontal lobes of the cerebral cortex during a neuronal firing are shown in Fig. 3. The signal is normalized to the maximum magnetic signal, specific to each set of the MPI setup and the magnetic properties of the nanoparticles. The region with a simulated neuronal firing is highlighted by the dotted circle. The synchronous firing is modeled to take place in a 2-mm local spot in the left pre-frontal region of the cortex. The images are taken at the initial instance after neuron firing. The neural oscillations in the brain are alpha rhythmic in the range of 8–12 Hz, with each oscillation period in the 100-ms range (Strijkstra et al., 2003; Li & Hopfield, 1989). No significant electric field variation as a result of such firing could be detected in the images taken with MNs. In contrast, with MENs, the signal in the region of the neuronal firing was different from that in the same region in its normal state (pre-firing) by approximately a factor of two, which reflected the fact that the locally generated electric fields were converted into local nanoparticles’ magnetization changes, which, in turn, could be detected by MPI as a change in the magnetic signal due to their ME effect. In other words, the local electric fields due to the synchronous firing modulated the magnetic image.

**Fig. 4:**
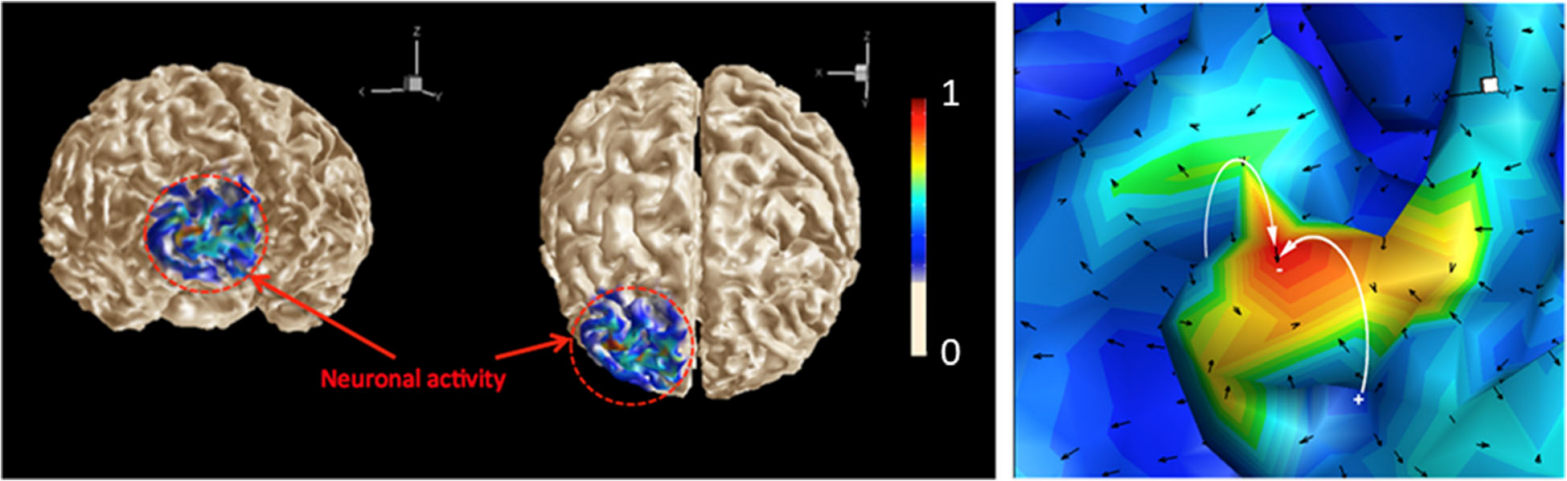
Normalized De-modulated MPI-MEN/MPI-MN images of two different angle views of frontal lobes. (Right) The insert shows a detailed normalized 3D field profile in the region of firing

The images of the same region obtained as a result of the demodulation of the MPI-MEN signal with the MPI-MN signal are shown in Fig. 4. According to this model, the de-modulated image shows the electric field map at the first instance after the neural firing in the highlighted region.

**Fig. 5:**
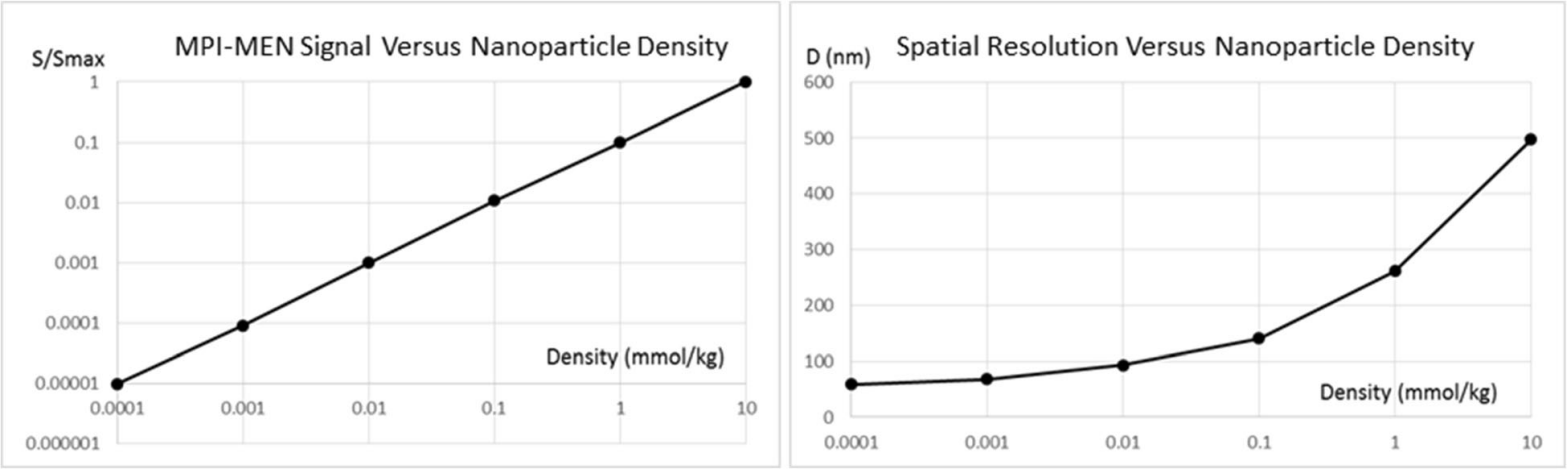
Nanoparticle density dependence of the signal and spatial resolution of MPI with 50-nm MENs

To understand the effect of the nanoparticle density on the image quality, the MPI’s signal and spatial resolution dependencies on the density are shown in Fig. 5. On the one hand, decreasing the distance between adjacent nanoparticles, in other words, with a density increase, should lead to a stronger signal. On the other hand, a shorter distance would lead to a stronger collective effect, which in turn interferes with the spatial resolution, which could explain the non-linear dependence of the spatial resolution on the density.

## Discussion

The objective of this computational study was to demonstrate the new capability of MENs, not provided by any other nanoparticle type, to map local intrinsic electric fields due to neural activity deep in the brain. The main hypothesis relied on the fact that MENs administrated into the brain served as energy-efficient local centers that coupled local intrinsic electric fields (due to neural activity) to an external magnetic imaging tool. As the imaging tool, the recently emerged approach of MPI was chosen because of its many advantages over the traditional MRI system, e.g., (i) a temporal resolution of faster than 1 μs, (ii) a weak or no dependence on the nuclear spin relaxation times and consequently, a superior sensitivity (arguably, at least three orders of magnitude better, depending on the specific MPI method), (iii) relaxed requirements on the background magnetic field strength and uniformity. With MPI, any magnetic nanoparticles, i.e. traditional MNs or recently developed MENs, could serve as imaging centers. Like the traditional MNs, if administrated intravenously, MENs could be steered through the blood system to the brain across the BBB via application of magnetic field gradients (Li & Hopfield, 1989). The approach of navigating MENs in the brain would be similar to that used in the conventional MRI with traditional MNs, e.g., SPIONs or gadolinium based nanoparticles, as contrast agents. After the nanoparticles enter the brain, they are not exposed to the relatively strong hydrodynamic force due to the blood circulation and therefore could be considered adequately stationary for the imaging duration. Assuming that there were enough MENs per each neuron to provide sufficient electric-field connectivity between the nanoparticles and the neuron, which was justified for the nanoparticle density range under study, each signal pixel in the MPI image reflected the local nanoparticle’s average magnetization i.e., S_MNI_ ~ M_rel_ (Fig. 2) (Probst et al., 2011). As expected, with the traditional MNs, the saturation magnetization didn’t depend on the electric-field microenvironment and therefore, represented mostly the brain’s structure (Fig. 2). In contrast, when MENs were used instead of MNs, because of the ME effect, the signal depended also on the local intrinsic electric field and therefore, reflected not only the local physical structure but also the local electric field due to the neural activity in the brain (Figs. 2 and 3). According to this model, the MPI-MEN signal was the result of the modulation of the structural image with the local electric field. Considering that MPI-MN provided mostly the structural image, similar to the one provided by conventional MRI, to obtain the electric field map of the brain, we de-modulated the MPI-MEN image with the equivalent MPI-MN image (Fig. 4). As for the spatial resolution, although the MPI signal significantly increased with increasing the nanoparticles’ density, the spatial resolution was limited to approximately 100 nm for 50-nm MENs, which could be explained by the collective dipole-dipole interaction effects at such high densities (Fig. 5). As for the temporal resolution, it is noteworthy that the MPI’s resolution (of < 1 μs depending on the MEN’s magnetic anisotropy) would be sufficient to monitor most neural activity in the brain in real time. Also, it could be noted that the studied electric-field mapping approach is relatively energy efficient and therefore would not cause any damaging thermal dissipation effects. To estimate the power that dissipates as a result of MPI-MEN imaging, we can make the following conservative back-of-the-envelope analysis. Assuming MENs are made of the popular coreshell composition of BaTiO_3_-CoFe_2_O_4_, with an atomic density of approximately 5 g/cc, a saturation magnetization of 10 emu/cc, and a coercivity field of 100 Oe, for a MENs’ net weight of 2.5 g, the nanoparticles would dissipate approximately 10^− 4^ J of energy in each M-H cycle. Then, taken an imaging frequency of 10 kHz, the dissipated power would be approximately 1 W, which, according to the thermal transport equation in the brain (Sotero & Iturria-Medina, 2011), would be significantly below the acceptable limit (of ~ 50 W) to avoid steady temperature rise by over 2 degrees. Last but not least, it is worth mentioning how the nanoparticles would be cleared from the brain post imaging. With the current rapid progress of nanotechnology and nanomedicine, eventually, biodegradable MENs will be developed, possibly made of biocompatible iron and/or carbon (Hong et al., 2012). However, there are several other alternatives which could be used in the not-so-distant future. For example, it has been shown that these nanoparticles are excreted naturally within 2 to 8 weeks depending on the nanoparticle’s size (Hadjikhani et al., 2017). Optionally, reversing magnetic field gradients, to ensure the maximum field is generated outside the brain region, could push the particles back to the blood circulation system and thus greatly accelerate the clearance process.

## Conclusions

In summary, this paper for the first time presented a theoretical study, supported through numerical simulations, which could pave the way to next-generation wireless electric-field mapping of the brain in real time. The main concept was based on integration of MENs with MPI to provide unique diagnostic and measurement capabilities. It was shown that placing MENs in the brain and then imaging them with MPI could allow to wirelessly monitor electric field activity deep in the brain at the sub-neuronal level in real time. In turn, such a capability could enable early screening and prevention of neurodegenerative diseases as well as pave the way to reverse engineering the brain.

## Abbreviations

BBB: blood brain barrier
BOLD: blood-oxygen-level dependent
CGAL: computer geometry algorithm library
CSF: cerebrospinal fluid
DfMRI: diffusion functional magnetic resonance imaging
dMRI: diffusion magnetic resonance imaging
DTI: diffusion tensor imaging
EEG: electroencephalography
EPSPs: excitatory postsynaptic potentials
FE: finite element
fMRI: functional magnetic resonance imaging
GM: grey matter
ME: magnetoelectric
MEG: magnetoencephalograph
MENs: magnetoelectric nanoparticles
MNs: magnetic nanoparticles
MPI: magnetic particle imaging
MRI: magnetic resonance imaging
PET: positron emission tomography
SPION: superparamagnetic iron-oxid nanoparticles
WM: white matter
